# Comparison of the Pharmacokinetic Properties of Hemoglobin-Based Oxygen Carriers

**DOI:** 10.3390/jfb8010011

**Published:** 2017-03-18

**Authors:** Kazuaki Taguchi, Keishi Yamasaki, Toru Maruyama, Masaki Otagiri

**Affiliations:** 1 Faculty of Pharmaceutical Sciences, Sojo University, 4-22-1 Ikeda, Nishi-ku, Kumamoto 862-0082, Japan; k-taguchi@ph.sojo-u.ac.jp (K.T.); kcyama@ph.sojo-u.ac.jp (K.Y.); 2 DDS Research Institute, Sojo University, 4-22-1 Ikeda, Nishi-ku, Kumamoto 862-0082, Japan; 3 Department of Biopharmaceutics, Graduate School of Pharmaceutical Sciences, Kumamoto University, 5-1 Oe-honmachi, Chuo-ku, Kumamoto 862-0973, Japan; tomaru@gpo.kumamoto-u.ac.jp; 4 Center for Clinical Pharmaceutical Sciences, Kumamoto University, 5-1 Oe-honmachi, Chuo-ku, Kumamoto 862-0973, Japan

**Keywords:** hemoglobin, oxygen carrier, liposome, pharmacokinetic

## Abstract

Hemoglobin (Hb) is an ideal material for use in the development of an oxygen carrier in view of its innate biological properties. However, the vascular retention of free Hb is too short to permit a full therapeutic effect because Hb is rapidly cleared from the kidney via glomerular filtration or from the liver via the haptogloblin-CD 163 pathway when free Hb is administered in the blood circulation. Attempts have been made to develop alternate acellular and cellular types of Hb based oxygen carriers (HBOCs), in which Hb is processed via various routes in order to regulate its pharmacokinetic properties. These HBOCs have been demonstrated to have superior pharmacokinetic properties including a longer half-life than the Hb molecule in preclinical and clinical trials. The present review summarizes and compares the pharmacokinetic properties of acellular and cellular type HBOCs that have been developed through different approaches, such as polymerization, PEGylation, cross-linking, and encapsulation.

## 1. Introduction

Oxygen (O_2_) is an essential element for most living organisms to maintain their biological activities. In the body, hemoglobin (Hb) in red blood cells (RBC) plays a central role in the delivery O_2_ from the lung to individual cells. As a result, the concentration of Hb in the blood circulation is maintained at 12–17 g/dL, in order to maintain a sufficient O_2_ supply. However, when unexpected massive bleeding caused by severe injury, an accident, or a surgical procedure occur, the Hb concentration drops to a lower level that may perturb various biological activities. In these cases, a RBC transfusion is the gold standard and is currently in widespread use for treating patients with massive hemorrhages. Nevertheless, RBC transfusions have some undesirable side effects associated with them, such as blood-type mismatching and infections caused by the presence of unrecognized pathogens. In addition, conventional RBC has a limited short storage period (up to 21–42 days in the world [1]) under cold storage conditions, and frozen storage remains unpractical. These problems make it difficult to use RBC, particularly in pre-hospital conditions and to maintain stable supplies of RBC in remote locations or in an emergency situation such as a disaster or a pandemic.

Researchers have attempted to prepare purified Hb from human- or animal-derived RBC in order to use them as an alternative to the conventional RBC transfusion. Purified Hb has some advantages, in that infectious viruses can be removed and they can be stored for much longer periods of time than RBC and that the removal of the cellular membrane allows for transfusions to patients without the need for any cross-match testing. However, the vascular retention of free Hb is not sufficiently long to fully achieve therapeutic effects. The reason for this is that Hb is rapidly cleared from the blood circulation via two pathways (as described in Section 2). To overcome these problems, attempts have been made to develop various types of modified Hb, which are referred to as acellular or cellular type Hb based O_2_ carriers (HBOCs) [2,3,4]. These HBOCs have a longer blood retention than free Hb by virtue of their original pharmacokinetic properties. Here, we briefly summarize the pharmacokinetic properties of acellular or cellular type HBOCs that have been used in research dealing with preclinical and clinical studies.

## 2. Stroma-free Hb

The Hb molecule is composed of four hemes (Fe-protoporphyrin IX) and a pair of identical αβ dimers (α_1_β_1_ and α_2_β_2_) that associate to form the Hb tetramer. Because of this, it is not possible for Hb to circulate in blood vessels for a long time (~0.5–1.5 h) [5]. Most of the Hb is packed inside RBCs in vivo because Hb can have several adverse effects. For example, Hb captures nitric oxide (NO), a vasorelaxation factor, from endothelial cells in blood vessels causing the induction of systemic or pulmonary hypertension [6]. Furthermore, although RBCs are equipped with an imbedded redox system and RBC contains enzymes that inhibit the formation of metHb or toxic substances, iron in the heme moiety of free Hb reacts easily with hydrogen peroxide or NO, to ultimately produce toxic substances such as oxoferryl Hb and transient free radical intermediates [6]. Endogenous- and exogenous-derived Hb is rapidly cleared from the body via two pathways. However, this degradation mechanism is prevented when Hb is packed in RBCs which remain in the blood circulation for longer periods of time and allows for a free exchange of O_2_. One of the clearing pathways is glomerular filtration in the kidneys [7]. When Hb is released into the blood circulation, the tetramer configuration of Hb spontaneously dissociates into two dimer configurations (α_2_β_2_→2αβ), which can easily pass through the glomerule because of its low molecular weight (approximately 32 kDa). Another clearing pathway is the identification by the Hb scavenger receptor (CD163) in the liver [8]. When Hb is released into the blood circulation, it is rapidly captured by the acute phase protein haptoglobin (Hp). The resulting Hb-Hp complex is removed from the circulation by CD163 recognition in the liver.

## 3. Recombinant Hb (rHb1.1, Optro^®^)

rHb1.1 is a molecular mutant human Hb produced in *Escherichia coli* in which the amino acid located at position 108 of both β chains has been changed (β108Asn→Lys) [9]. Furthermore, two α globin chains are genetically fused with a glycine bridge between the C-terminal and the N-terminal amino acid of each α globin to prevent the dissociation into two dimers (α_2_ β_2_→2αβ). The half-life of the rHb1.1 molecule in vivo is prolonged by 3–4 fold compared to native Hb by virtue of the two α globin units being fused [9]. In a clinical trial (Phase I), the half-life of rHb1.1 was found to increase (2.4–18.9 h) with increasing dose (0.015–0.32 g/kg) [10]. Although rHb1.1 actually advanced to Phase II, it was discontinued due to adverse effects [11]. A second generation product, rHb2.0, has been developed and its efficacy is being evaluated in preclinical trials [12,13]. Furthermore, Marquardt and colleagues prepared di-Hb in which two rHb1.1 units are covalently cross-linked by gene fusion using a peptide linker and they also prepared tetra-Hb that is chemically cross-linked with bismaleimidohexane between a cysteine residue into di-Hb [14]. A pharmacokinetic study in rats demonstrated that the half-life of di-Hb and tetra-Hb are approximately 1.4 and 1.6 times longer than that of rHb1.1 [14,15].

## 4. Acellular Type HBOCs

Since the 1980s, many types of acellular HBOCs, including polymerized Hb, intramolecularly cross-linked Hb, and poly(ethylemeglycol)-conjugated (PEG-conjugated) Hb, have been developed and examined in preclinical and clinical trials [3,16]. Their modification (PEGylation, cross-linking, and polymerization) was found to prolong vascular retention compared to free Hb [17]. In this section, we introduce some of the developed acellular HBOCs and summarize their pharmacokinetic results obtained in preclinical and clinical trials.

### 4.1. HbNFPLP

2-nor-2-formylpyridoxal 5′-phosphate (NFPLP) can form a cross link between the two β chains of Hb, resulting in an intramolecular cross-linked form of Hb [18]. Using this characteristic, Bakker et al. prepared an Hb that was modified by cross-linking with NFPLP (HbNFPLP) in the 1980s [19,20]. They investigated the vascular retention of HbNFPLP in blood exchange model rats and rabbits using a unique method in which a 1:1 mixture of unmodified Hb and HbNFPLP were transfused [21]. This method enables the blood retention between unmodified Hb and HbNFPLP to be compared in the same animals. As a result of this study, the half-life of HbNFPLP was found to be approximately 3 and 2.7 times longer than that of unmodified Hb in rats and rabbits, respectively (1.1 h and 3.2 h in rats, 2.7 h and 7.2 h in rabbits, for unmodified Hb and HbNFPLP, respectively). Similar results were reported by another group using tritium (^3^H) labeled HbNFPLP in healthy rats [22]. Furthermore, they evaluated the difference in biodistribution between unmodified Hb and HbNFPLP using a technetium 99m (^99m^Tc) technique [23]. Although the ^99m^Tc activity was mainly detected in the liver and kidney at 2 h after ^99m^Tc-labeled HbNFPLP administration (5% and 4% of the dose in the kidney and liver, respectively), it was much less than that for the ^99m^Tc-labeled unmodified Hb (15% and 6% of the dose in the kidney and liver, respectively). In addition, light microscopy observations after staining with diaminobenzidine showed that strong Hb accumulations were seen in the lumina of many proximal and distal tubules after the administration of unmodified Hb, but rarely after the administration of HbFLPLP. These results indicate that intramolecular cross-linking prevents glomerular filtration resulting from maintaining the tetramer configuration of Hb. These pharmacokinetic results are summarized in Table 1.

### 4.2. Diasprin Cross-Linked Hb (DCLHb, HemAssist^®^)

Diasprin cross-linked Hb is an intramolecular cross-linked HBOC (DCLHb), in which human Hb is cross-linked between the α-chain with bis(3,5-dibromosalicyl)fumarate. In the early 1980s, researchers began working on the development of this product. Its pharmacokinetic properties were evaluated in preclinical and clinical trials (Table 1). Keipert et al. reported on its pharmacokinetic properties, such as plasma retention, tissue distribution, and excretion, in a rat model of exchange transfusion using carbon 14 (^14^C)-labeled DCLHb (the total administered dose was 2400 mg/kg) [24,25]. They demonstrated that the half-life for DCLHb was 5 h and tissues containing high ^14^C radioactivities were the kidney, spleen, bone marrow, and liver. In addition, they monitored ^14^C radioactivities in tissues for as long as 14 days, and demonstrated that 2 weeks were required for the metabolic degradation and elimination of a large dose of DCLHb. The pharmacokinetic properties of DCLHb have also been evaluated in a rat model of sepsis [26] and hepatic cirrhosis [27]. In a human study, the half-life of DCLHb was 2.5–3.3 h in healthy volunteers [28] and 2.1–4.3 h in patients who were receiving chronic hemodialytic therapy [29] at a dose of 25, 50, and 100 mg/kg. The changes in the plasma concentration of DCLHb in large blood loss surgical patients and patients with cardiac surgery have also been investigated [30,31]. However, its development for clinical use was stopped in 1999 [3].

### 4.3. Polymerized Bovine Hb (HBOC-201, Hemoglobin Glutamer-250 (Bovine), Hemopure^®^)

HBOC-201 (hemoglobin glutamer-250 (bovine), Hemopure^®^) is bovine Hb that has been polymerized with glutaraldehyde. Although a number of preclinical studies of HBOC-201 have been performed and its efficacy has been demonstrated [32], to best of our knowledge, pharmacokinetic studies of HBOC-201 in preclinical trials are limited, due to the fact that the half-life of HBOC-201 is around 22 h in a 40% hemorrhagic shock model of a Yucatan mini-pig [33] (or have not been published). On the other hand, the safety and efficacy of HBOC-201 has been examined in more than 20 clinical trials [34], some in which the half-life of HBOC-201 in humans were reported (Table 2). Hughes et al. studied the pharmacokinetics of HBOC-201 in normal healthy men who underwent a 15% blood volume phlebotomy, followed by volume replacement with lactated Ringer’s solution, and then an infusion of HBOC-201 with dose-escalation (16.5–45 g Hb/person) [35,36]. In these studies, a dose-dependent increase in the plasma Hb peak was observed and the half-life was found to be 16–20 h. Furthermore, the half-life of HBOC-201 was similar in men and women (men; 20.0 ± 2.4 h, women; 20.3 ± 1.2 h) [37]. In addition, another prospective, randomized study in which HBOC-201 was administered to patients undergoing a liver section at a dose of 400 mg/kg showed that the half-life of HBOC-201 in these patients was about 8.5 h [38]. These long half-lives of HBOC-201 can be attributed to preventing filtration from glomerul due to its increase in molecular weight. In fact, no detectable hemoglobinuria were observed in clinical trials [37]. HBOC-201 is currently approved for the treatment of anemia and for use during surgery in South Africa since 2001 [39,40].

### 4.4. Hb-raffimer (Hemolink^®^)

Hb-raffimer (Hemolink^®^) is an o-raffinose cross-linked and oligomerised form of human Hb. The molecular weight of Hb-raffimer ranges from 32 kDa to 500 kDa, indicating that a Hb-raffimer solution is a mixture that contains heterogeneous Hb up to nanomer-sized molecules [41]. Hsia et al. investigated the pharmacokinetic difference of Hb-raffimer among different molecular weights using an ^3^H labeled Hb-raffimer in a rat model of isovolemic 50% exchange transfusion [42]. Although the half-life of the total Hb-raffimer was around 10 h, the half-life values for the individual molecular weight of Hb-raffimer were prolonged, ranging from 0.6 h to 13 h with increasing molecular weight. Since the pharmacokinetic evaluation in healthy rats indicated that the Hb-raffimer was mainly distributed in the kidney and liver [43], they determined the hepatic and renal distribution of the Hb-raffimer with different molecular weights. The distribution in the kidney was decreased with increasing molecular weight of Hb-raffimer, while the distribution in liver was increased with increasing molecular weight of Hb-raffimer. Another study reported the half-life of Hb-raffimer preparations with different molecular weights in a healthy dog that was administered a 25% top load infusion of Hb-raffimer [44]. Along with results obtained in an isovolemic 50% exchange transfusion model rat, the half-life values of individual molecular weights of Hb-raffimer were prolonged with increasing molecular weight (total Hb-raffimer; 25.4 h, Hb-raffimer (64 kDa); 11 h, Hb-raffimer (128 kDa); 25 h, Hb-raffimer (>128 kDa; 42 h). In addition to animal studies, Carmichael et al. reported that the half-life of total Hb-raffimer was approximately 14 h, and that for the oligomer (>64 kDa) it was 18–20 h in Phase I study [45]. Although the Hb-raffimer has been evaluated in Phase III trials [46,47,48], its development for clinical use has been suspended.

### 4.5. Pyridoxal PolyHb (PolyHeme^®^)

Pyridoxal PolyHb is a chemically modified human Hb solution. The developers began developing this preparation in 1969 in conjunction with the U.S. Army, and Northfield Laboratories then began work on its development for clinical use. This product is pyridoxylated to increase O_2_ affinity (P_50_) and polymerized with glutaraldehyde. Although pyridoxal PolyHb has already completed Phase III trials [49,50], as far as we know, reports of its pharmacokinetic properties are limited. Sehgal et al. compared the vascular retention between stroma free Hb and pyridoxal PolyHb in adult baboons (exchange transfusion model) [51]. After a 900 mL partial exchange, the half-life of the stroma free Hb and pyridoxal PolyHb were determined to be around 6 h and 46 h, respectively. In addition, some articles reported that the half-life of pyridoxal PolyHb in humans is around 24 h based on data obtained from clinical trials [49,52]. Unfortunately, the development of pyridoxal PolyHb for clinical use was stopped in 2009 [3].

### 4.6. MP4 (Hemospan^®^)

MalPEG-hemoglobin (MP4) is maleimide-PEG modified human Hb developed by Sangart Inc. (San Diego, CA, USA). The molecular weight of MP4 is about 100 kDa, because a mean of 7–8 PEG_5000_ is conjugated to one human Hb [53]. Animal studies with MP4 indicate that the half-life of MP4 is about 24 h in rats at a dose of 2600 mg/kg and about 18 h in monkeys at a dose of 840 mg/kg [54,55] (Table 3). MP4 has been undergoing clinical safety evaluations since 2002. A phase I study indicated that the half-life of MP4 in healthy volunteers was 66.2 and 42.8 h at a dose of 50 mg/kg and 100 mg/kg, respectively [56]. Interestingly, serum Hp completely disappeared immediately after MP4 administration, suggesting that MP4 was captured by Hp and eliminated via CD163, similar to endogenous Hb [8], albeit PEG would be expected to prevent the interactions between Hp and Hb. Olofsson et al. reported two randomized Phase II studies that were carried out to evaluate the safety and efficacy of MP4 at a dose of 100–500 mg/kg during spinal anesthesia in patients who were undergoing orthopedic surgery [57,58]. The half-life of MP4 in these patients ranged from 14 to 23 h, which was prolonged with increasing dose. Urinary Hb measured by the dipstick method showed an equivalent value to that for control patients [57,58]. In addition, Hp levels fell to 0 in these patients as well as healthy volunteers [57]. Taking these findings into consideration, MP4 appears to be mainly eliminated via CD163 after binding with Hp, even in orthopaedic surgery patients with spinal anesthesia. 

### 4.7. PEG-Hb™

Enzon Inc. (Piscataway, NJ, USA) developed a PEG conjugated bovine Hb (PEG-Hb™) in which each bovine Hb is modified with approximately 12 succinimidyl carbonate PEG_5000_. PEG-Hb™ has been previously reported to be safe and effective in a number of animal species models [59,60,61,62]. Changes in the plasma concentration of PEG-Hb™ after a top-load infusion (25 mL/kg) was examined in rats and rabbits, and the findings showed that the mean half-life was approximately 17 h and 43 h in rats and rabbits, respectively [61,63]. In addition, PEG-Hb™ was shown to have a half-life of 15 h and 58 h in rat and dog models of 30% blood volume exchange transfusion [62,64]. Furthermore, PEG-Hb™ showed a longer half-life than native Hb in a canine hemorrhagic shock model [65] These long half-lives, as summarized in Table 3, indicate that PEG modification can prevent excretion via glomerular filtration due to the increase in overall molecular weight. In fact, less hemoglobinuria were observed in animals that had been injected with PEG-Hb™ than those injected with native Hb [61,63]. Although PEG-Hb™ had earlier proceeded to Phase Ib, its development for clinical use was stopped more than 20 years ago [3].

### 4.8. SB-1

SunBio1 (SB-1) is a PEG-conjugated bovine Hb [66]. The purified Hb derived from bovine Hb was reacted with activated methoxy PEG_5000_ to prepare SB-1 in which each bovine Hb is PEGylated with an average of 8 PEG. The resulting SB-1 has a molecular weight of 104.5 kD with a size of 30–50 nm. Pharmacokinetic studies of SB-1 have only been evaluated in animals (Table 3). A pharmacokinetic study of SB-1 using iodine125 (^125^I)-labeled SB-1 showed that the half-life of SB-1 is 9.6 h and 10.6 h in male rats at a dose of 5 mL/kg and 12.5 mL/kg, respectively [67]. Furthermore, pharmacokinetic analyses after single and multiple injections in male beagle dogs showed that the half-life for a single injection increased with the dose (7.7 h, 9.7 h, and 17 h for 2.5 mL/kg, 5 mL/kg, and 10 mL/kg, respectively). On the other hand, the half-life values for multiple injections were about two times higher than that of the single dose in male beagle dogs. However, no significant difference in pharmacokinetic properties was found between male and female beagle dogs [68].

## 5. Newer Generated Acellular Type HBOCs

In 2008, Natanson et al. reported the results of a meta-analysis that used 16 randomized controlled trials of five types of acellular HBOCs (DCLHb, HBOC-201, Hb-raffimer, pyridoxal PolyHb, and MP4) [69]. This report concluded that using acellular HBOCs is associated with a significantly increased risk of death and myocardial infract compared to a control solution. After releasing this meta-analysis, the development of new acellular type HBOCs was accelerated [70]. In this section, we discuss the development of new types of acellular HBOCs and summarize their pharmacokinetic results obtained in preclinical and clinical trials.

### 5.1. Oxy Vita-Zero-Link Polymerized Hb (Oxy Vita^®^Hb)

Oxy Vita-Zero-Link polymerized Hb is a novel type of acellular HBOC, the design and approach of which was initially carried out by Dr. Bucci using zero-linked polymerized technology [71]. Each intramolecular cross-linked bovine Hb (cross-linker: bis (3,5 dibromosalicyl-adipate)) is polymerized via a stable amide bond between the carboxylate groups of C-terminal or side chains of glutamic acid (Glu) and aspartic acid (Asp) and N-terminal amino groups or amino side chains of the lysyl residue [72]. The mean molecular weight of Oxy Vita^®^Hb is about 17 MDa, substantially larger than that for other acellular HBOCs, and its physicochemical properties are also well defined [73,74,75,76]. Matheson et al. reported on the intravascular retention of Oxy Vita^®^Hb in rats and cats [77]. The half-life of Oxy Vita^®^Hb was approximately 7 h and 10 h in rats and cats, respectively. Furthermore, Oxy Vita^®^Hb with a large radius showed an 8–12 times longer half-life than that with a small radius [72]. This product is still in the preclinical trial stage of development. The pharmacokinetic data in clinical trials are being awaited.

### 5.2. PEGylated carboxyHb (SANGUINATE™, MP4CO)

SANGUINATE™ is a PEGylated bovine carboxyHb in which each bovine Hb molecule is modified with PG-succinimidyl carbonate PEG_5000_. This product is expected to avoid the problems associated with NO scavenging and auto-oxidation, and functions as both a carbon monoxide (CO) releasing molecule and an O_2_ transfer agent [78]. No adverse effects were observed at a dosage level of 1200 mg Hb/kg in monkeys, 1600 mg Hb/kg in pigs, and 2400 mg Hb/kg in rats [78,79,80]. From the pharmacokinetics viewpoint, changes in plasma carboxyHb in the rat were evaluated [81]. Plasma carboxyHb increased to around 6% at 1 min after the administration and then gradually decreased to 1% at 2 h after administration. In a human study (Phase I), SANGUINATE™ was found to be safe and well tolerated in healthy volunteers, and its half-life was dose dependent and ranged from 7.9 h to 13.8 h [82]. Interestingly, the half-life of SANGUINATE™ at a dose of 160 mg Hb/kg was prolonged in stable sickle cell anemia patients (about 20 h) compared to that in healthy volunteers (13.8 h) [83]. Although two cases where patients received SANGUINATE™ under emergency conditions were reported [78], large scale clinical trials in patients will be needed prior to clinical applications.

MP4 saturated with CO (MP4CO) is a PEGylated human Hb saturated with CO and an O_2_ therapeutic that has shown potential in preventing and reversing RBC sickling. The efficacy of MP4CO, including anti-adhesive, anti-inflammatory, anti-oxidant, and anti-apoptotic properties was demonstrated in a murine model of sickle cell disease, subarachnoid hemorrhage, and myocardial infract [84,85,86]. MP4CO has already completed safety and tolerability studies in patients with sickle cell disease (Phase Ib) [87]. The carboxyHb level was observed to be dose-dependent, and then became normalized to pre-dosing levels by 8 h. Unfortunately, to the best of our knowledge, this is only report related to the pharmacokinetics of MP4CO. However, since the basic structural and physicochemical properties of MP4CO are very similar to those for MP4, its pharmacokinetic properties would also be similar to MP4 as shown in Section 4.6 in this article. 

### 5.3. Hb–Albumin Clusters (HemoAct™)

Komatsu et al. recently developed a novel type of acellular HBOCs, referred to as Hb–albumin clusters [88]. The Hb–albumin cluster is composed of one Hb in the center and three or four human serum albumins (HSA) in the periphery, and was prepared by a covalent linkage between the Cys34 residue of HSA and the surface Lys amino groups of Hb via a heterobifunctional cross-linker. HemoAct showed good blood compatibility in vitro, and it induced neither hypertension nor toxicity in vivo [89]. Komatsu and colleagues prepared three types of Hb-HSA clusters, Hb-HSA_3_ (Hb was enclosed by three HSA molecules), Hb-HSA_4_ (Hb was wrapped by four has molecules), and XLHb-HSA_3_ (intramolecularly crosslinked Hb was enclosed by three HSA molecules), and compared their pharmacokinetic properties [89,90]. The half-lives of all types of Hb-HSA clusters were more than 20-times and 1.5 times longer than that of Hb and has, respecctively. This is why Hb-HSA clusters prevent not only extravasation through the vascular endothelium, but also filtration via the renal glomerulus. When the half-life among Hb-ablumin clusters was compared, the half-life of Hb-HSA_4_ was comparable to that for Hb-HSA_3_ in spite of the large molecular weight. This suggests that an increase in the molecular weight of the cluster (number of albumin) has no influence on extending the circulation time for the preparation. In fact, a similar phenomenon was observed in a study using albumin clusters [91]. On the other hand, the half-life of XLHb-HSA_3_ was decreased compared to Hb-HSA_3_. The reason for this phenomenon is unclear but it appears to be attributable to differences in the morphologies of the clusters.

In addition to the Hb-Albumin clusters mentioned above, Komatsu’s group also prepared a Hb-HSA_3_ cluster in which a Pt nanoparticle was incorporated into the exterior HSA unit of the cluster, to add antioxidant activity, which would be expected in many clinical situations involving ischemia-reperfusion injuries [92]. Furthermore, Oxyglobin^®^ (polymerized bovine Hb) is available for the treatment of anemia in dogs [93]. Yamada et al. are attempting to prepare new Hb-Albumin clusters using Hb and albumin derived from different mammalian species for use in veterinary medicine [94]. Their unique ideas may be able to realize HBOCs for not only human but also for animal pets.

## 6. Cellular Type HBOCs

Historically, many types of cellular type HBOCs have been developed since 1957 (see the review by Sakai et al. [95,96]). The concept of cellular types of HBOCs is to mimic the cellular structure of the RBC, which can retard binding between Hb and gaseous messenger molecules (NO and CO) in blood vessels. Additionally, the cellular type of HBOCs can also prevent the dissociation of tetramic Hb subunits into two dimers. These effects would be expected to shield against some adverse effects derived from Hb such as hypertension and renal failure. From the pharmacokinetic viewpoint, cellular type HBOCs showed different properties than acellular type HBOCs because their pharmacokinetic properties would be more strongly influenced by the characteristics of the nanoparticles than that of Hb. The current tendency for cellular type HBOCs is liposome-encapsulated Hb composed of phospholipids, cholesterol, fatty acids, etc. In the following section, we summarize the pharmacokinetic properties of two different kinds of liposome-encapsulated Hb, which have the potential to proceed to the clinical trial stage.

### 6.1. TRM-645 (Neo Red Cells)

Liposome encapsulated Hb (cord name: TRM-645) was developed by the Terumo Corp. (Tokyo, Japan) in 1995 and its physicochemical properties are well defined [97]. The mean diameter of TRM-645 particles ranges from 200 to 250 nm and encapsulates purified and highly concentrated human Hb and inositol hexaphosphate as an allosteric effector in a multi-lamellar lipid membrane. The liposomal surface is further modified by PEG5000-DSPE, which is intended to enhance the stability of TRM-645 during both storage and in the blood circulation by preventing aggregation [98,99]. Furthermore, preclinical studies have been performed and the findings indicate that the product is efficacious and safe as a RBC substitute in some animal species including the rat, dog, and monkey [97,100]. 

The pharmacokinetic properties of TRM-645 were evaluated using rats and cynomolgus monkeys. Kaneda and co-workers administered TRM-645 to healthy rats and monkeys at a dose of 20 mL/kg and evaluated the changes in its concentration in the blood circulation using Enzyme-Linked Immuno Sorbent Assay (ELISA) that specifically reacts with human Hb [97]. As a result, the half-life in rats was found to be approximately 10 h and human Hb reached a non-detectable level at 72 h after administration. On the other hand, the half-life in monkeys was much longer than that in rats (half-life: 70 h), and human Hb was still observed in the blood circulation at 72 h after administration. These results for different animals suggest a longer half-life in humans. In addition, lipid particles were observed in reticuloendothelial organs such as the spleen, liver, and bone marrow in repeated-dose toxicological studies in rats and monkeys (20 mL/kg), which indicate that TRM-645 is likely captured and degraded by the mononuclear phagocyte system (MPS) as well as other liposome preparations [101]. However, information regarding the pharmacokinetic properties of TRM-645 are limited to its retention in the blood circulation and its distribution in reticuloendothelial organs in a few animal species (Table 4). Further study will clearly be needed to determine the detailed pharmacokinetic properties such as the metabolic and accumulative properties of TRM-645 and its components in both healthy and critical conditions in which TRM-645 would be expected to be used. 

### 6.2. Hemoglobin-Vesicles (HbV)

Since around 1985, Hemoglobin-vesicles (HbV) have been developed by Tsuchida’s group at Waseda University. HbV are promising cellular types of HBOCs, in which concentrated human Hb is encapsulated and the particle surface is covered with PEG. HbV has been demonstrated to have pharmacological effects that are comparable to RBCs in hemorrhagic shock model animals [105,106,107,108]. We have accumulated data regarding the pharmacokinetic profiles of HbV in detail using healthy animals, as shown in Table 4. Based on these data, the half-life of HbV in humans was estimated to be approximately 3 to 4 days, based on the use of allometric equations [109], which is, unfortunately, much shorter than RBC (around 120 days). Detailed data on the pharmacokinetics of HbV in healthy animals are not reviewed here, since a review of this subject was previously published by our group [110].

We also investigated the pharmacokinetic profile of HbV in various animal models with some disorders that mimic a clinical setting (Table 5). In a previous study, the pharmacokinetic profiles of HbV under hemorrhagic shock conditions, in which HbV could be used, were investigated using a rat model and the results were compared to healthy conditions [109]. The half-life of HbV in hemorrhagic conditions was about 18 h, which was 0.6 times shorter than healthy conditions (about 30 h). Furthermore, our group investigated the pharmacokinetic properties of HbV using chronic liver cirrhosis model rats with fibrosis [111]. Interestingly, the pharmacokinetic parameters (the total clearance, hepatic distribution of HbV, and the amount of cholesterol excreted in feces) were negatively correlated with plasma aspartate aminotransferase levels, which reflected hepatic injury. The detailed pharmacokinetic properties of HbV in hemorrhagic shock and chronic liver failure conditions were reviewed elsewhere (see [110]).

The disposition of HbV for use in pregnant mothers and fetuses has also been investigated [112]. Pregnant rats were given daily repeated injections of HbV (2000 mg/kg) from days 16 to 22 of pregnancy. In a non-labeled study, the concentrations of lipids, bilirubin, and ferric iron (Fe^3+^), which should be released as the result of the decomposition of Hb, were not significantly changed after the 7 day daily repeated injections of HbV. In addition, histological evaluations showed a slight detection of human Hb and hemosiderin in the maternal liver, spleen, kidney, and in the junctional zone or labyrinth of the placenta, but was not detected in any organs of the fetus. Furthermore, a pharmacokinetic study using ^125^I-HbV showed that HbV was distributed mainly in the spleen and liver in rat mothers, but was not distributed in the fetal organs.

We previously investigated the pharmacokinetic properties of HbV in Apo E deficient mice (B6.KOR/StmSlc-Apoe*^shl^* mice) because of the concern that the disposition of HbV, especially lipid components, may be altered due to their poor metabolic and excretion profiles [113]. Biological parameters which are related to lipid metabolites of HbV such as cholesterol were temporarily increased after an HbV injection in B6.KOR/StmSlc-Apoe*^shl^* mice, but completely recovered to basal levels at 7 days after the injection. In addition, pharmacokinetic analyses using ^3^H-HbV in B6.KOR/StmSlc-Apoe*^shl^* mice clearly showed that the HbV had disappeared from all organs within 14 days after the HbV injection.

Taking the above findings into consideration, HbV is promptly metabolized and excreted under not only healthy conditions but also under any pathological conditions, suggesting that HbV has favorable pharmacokinetic properties as a RBC substitute. Our group is currently planning to draft a protocol for a clinical study (Phase I) according to the guidance for O_2_ carrier products by The Society of Blood Substitutes Japan [114]. In addition to functioning as a RBC substitute, we and other colleagues recently showed another potential use for HbV based on its O_2_ and carbon monoxide transport characteristics, which lead us to expect that it has a variety of other applications [115,116,117,118,119]. Thus, HbV has enormous potential for clinical applications, and the first clinical trials of HbV toward realization in clinics are being awaited.

## 7. Conclusions

A number of HBOCs, as described above, have been developed and their efficacy, safety, and pharmacokinetics have been evaluated in preclinical and clinical trials. Recently, Estep TN pointed out a few pitfalls with respect to the circulatory retention of HBOCs from the viewpoint of (i) methods of analysis; (ii) incorrect pharmacokinetic results due to changes in blood volume; (iii) experimental animal models and (iv) functional half-life [121]. Unlike other drugs, since the dosage volume of HBOCs is more than a hundred times larger than that of other drugs, the pharmacokinetic parameters cannot be calculated by the usual pharmacokinetic analysis methods. It may be necessary to establish a standardized pharmacokinetic analysis method for HBOCs in the future.

Since pharmacokinetic properties including blood retention depend on the dose, animal species, molecular weight, and pathological conditions, it is difficult to compare the pharmacokinetic properties and to determine the merits and drawbacks among HBOCs with different modifications from pharmacokinetic results in different experimental conditions. In any event, most acellular and cellular HBOCs showed superior therapeutic effects compared to Hb because of their longer half-life. However, nobody has yet produced HBOCs that possess a comparable half-life to RBC. In addition, although the inhibition of methemoglobin formation (functional half-life) is also important for HBOCs to function as a RBC substitute, it was quite difficult to completely reproduce the redox system of RBCs in HBOCs due to the complex biological systems of RBC. Therefore, from the viewpoint of the pharmacokinetic properties of HBOCs, HBOCs are expected to function as O_2_-bridge agents before RBC transfusions, rather than as an entire RBC alternative. Needless to say, the realization of RBC substitutes is urgent, considering the medical and social problems that now face society. In the near future, it is expected that acellular or cellular HBOCs will be clinically developed for use in patients who need oxygen carrier support until appropriate units are found.

## Figures and Tables

**Table 1:** Pharmacokinetic summary of intramolecular cross-linking HBOCs.

Characteristic	DCLHb	NbNFPLP
**Modification**		
cross-linker	bis(3,5-dibromosalicyl)fumarate	2-nor-2-formylpyridoxal 5′-phosphate
cross-linked amino acid	Lys99 residue in the two α-chain	Val1-Lys82 in the two β-chain
**Pharmacokinetic studies**		
Animal	**Blood exchange model–rat [24,25]**	**Healthy rat [22,23]**
	Half-life: 5 h (2400 mg/kg)	Half-life: 2.7 h (145 mg/kg)
	Distribution: kidney, spleen, bone marrow, liver	Distribution: kidney, spleen, liver
	**Sepsis model–rat [26]**	**Blood exchange model–rat [21]**
	Half-life: 4.2 h (300 mg/kg)	Half-life: 3.2 h (~2000 mg/kg)
	**Hepatic cirrhosis model–rat [27]**	Less excretion into urine than Hb
	Half-life: 4.7 h (400 mg/kg)	**Blood exchange model–rabbit [21]**
		Half-life: 7.2 h (1400 mg/kg)
Human	**Healthy volunteer [28]**	No clinical study
	Half-life: 2.5–3.3 h (25, 50, 100 mg/kg)	
	**Hemodialysis patient [29]**	
	Half-life: 2.1–4.3 h (25, 50, 100 mg/kg)	
	**Large blood loss surgical patient [31]**	
	Half-life: 10 h (658–1500 mg/kg)	
	**Cardiac surgery patient [30]**	
	Half-life: ~24 h (~7500 mg/person)	

HBOCs: Hb based O_2_ carriers, DCLHb: Diasprin cross-linked Hb, NFPLP: 2-nor-2-formylpyridoxal 5′-phosphate

**Table 2:** Summary of the pharmacokinetics of polymerized HBOCs.

Characteristic	HBOC-201	Hb-Raffimer	Pyridoxal PolyHb
**Modification**			
Species of Hb	bovine	human	human
Modification	Glutaraldehyde polymerization	Cross-linking with o-raffimer	Pyridoxylation and glutaraldehyde polymerization
Molecular weight	Average; 250 kDa	32–500 kDa	No reported
**Pharmacokinetic studies**			
Animal		**Healthy rat [43]**	
		Half-life: 5 h (65 mg/kg)	
	**Hemorrhagic shock model–pig [33]**	**Healthy dog [44]**	**Blood exchange model–baboon [51]**
	Half-life: 22 h (1300–3200 mg/kg)	Half-life: 25.4 h (1.8 g/kg)	Half-life: 46 h (90 g/ baboon?)
		**Blood exchange model–rat [42]**	
		Half-life: 10 h (dose: unknown)	
Human	**Healthy volunteer [35,36]**		
	Half-life: 16–20 h (16.5–45 g/person)	**Healthy volunteer [45]**	**Healthy volunteer (?) [49,52]**
	**Patient undergoing a liver section [38]**	Half-life: 18–20 h (25–600 mg/kg)	Half-life: ~24 h (dose: unknown)
	Half-life: 8.5 h (400 mg/kg)		

**Table 3:** Summary of the pharmacokinetics of PEGylated HBOCs.

Characteristic	MP4	PEG-Hb	SB-1
**Modification**			
Species of Hb	human	bovine	bovine
Number of PEG	7–8	12	8
Size of PEG (Da)	5000	5000	5000
**Pharmacokinetic studies**			
Animal		**Healthy rat [61]**	**Healthy rat [67]**
		Half-life: 17 h (25 mL/kg)	Half-life: 9.6–10.6 h (5, 12.5 mL/kg)
		**Healthy rabbit [63]**	**Healthy dog [68]**
		Half-life: 43 h (25 mL/kg)	*Single injection (Male)*
	**Healthy rat [54]**	**Blood exchange model–rat [62]**	Half-life: 7.7–17 h (2.5–10 mL/kg)
	Half-life: 24 h (2600 mg/kg)	Half-life: 15 h	*Multiple injection (Male*)
	**Healthy monkey [55]**	No hemoglobinuria	Half-life: 16.7–38 h (2.5–10 mL/kg)
	Half-life: 18 h (840 mg/kg)	**Blood exchange model–dog [64]**	*Single injection (Female)*
		Half-life: 58 h	Half-life: 6.2–14.4 h (2.5–10 mL/kg)
		**Hemorrhagic shock model–dog [65]**	*Multiple injection (Female)*
		Half-life: 18 h	Half-life: 17.7–28.5 h (2.5–10 mL/kg)
Human	**Healthy volunteer [56]**	No clinical study	No clinical study
	Half-life: 42.8–66.2 h (50–100 mg/kg)		
	**Patient with orthopedic surgery [57,58]**		
	Half-life: 14–23 h (100–500 mg/kg)		
	No hemoglobinuria		

MP4: MalPEG-hemoglobin, PEG-Hb: PEG conjugated bovine Hb, SB-1: SunBio1

**Table 4:** Preclinical pharmacokinetic studies of cellular type HBOCs in healthy animals.

Characteristic	TRM-645	HbV
**Property**		
Mean diameter (nm)	200–250	250–280
Hb concentration (g/dL)	6	10
Lipid composition	Soybean hydrogenated phosphatidylcholine, cholesterol, stearic acid, PEG_5000_-DSPE	DPPC, cholesterol, DHSG, PEG_5000_-DSPE
**Pharmacokinetic studies**		
	**Rat [97]**	**Mouse [102]**
	Half-life: 10 h (20 mL/kg)	Half-life: 20 h (1400 mg/kg)
	No detectable Hb in circulation at 72 h after administration	Distribution: spleen, liver
		Excretion: degraded Hb into urine lipid composition into feces
	**Monkey [97]**	**Rat [102]**
	Half-life: 70 h (20 mL/kg)	Half-life: 30 h (1400 mg/kg)
	Detectable Hb in circulation at 72 h after administration	Distribution: spleen, liver
		Excretion: degraded Hb into urine lipid composition into feces
		**Rabbit [103]**
		Half-life: 63 h (1400 mg/kg)
		**Monkey [104]**
		Half-life: 47–72 h (1400 mg/kg)

DPPC, 1,2-dipalmitoyl-sn-glycero-3-phosphatidylcholine; DHSG, 1,5-*O*-dihexhadecyl-*N*-succinyl-l-glutamate; PEG5000-DSPE, (*N*-[monomethoxypolyethyleneglycol-carbamyl] distearoylphosphatidyl-ethanolamine).

**Table 5:** Pharmacokinetic studies of HbV in pathological model animals.

Model	Species	Dose	Pharmacokinetic Characteristic
Hemorrhagic shock [109]	Rat	1400 mg/kg	(i) half-life of HbV in hemorrhagic shock rats was reduced compared with healthy rats (ii) HbV was mainly distributed in liver and spleen as well as healthy rats
Hepatic cirrhosis [111,120]	Rat	1400 mg/kg	(i) the retention of HbV in circulation were prolonged in the chronic cirrhosis rat model compared to healthy rats (ii) the hepatic distribution of HbV was decreased in the chronic cirrhosis rat model compared to healthy rats (iii) the amount of lipid component (cholesterol) in feces was less in the chronic cirrhosis rat model than in healthy rats
Hyperlipidemia [113]	Mouse	2000 mg/kg	(i) HbV was cleared from blood circulation within 3 days after injection (ii) HbV was mainly distributed were the liver and spleen (iii) Lipid component had cleared from each organ by 14 days after injection
Pregnancy [112]	Rat	1400 mg/kg	(i) HbV was distributed mainly in spleen and liver in rat mothers (ii) no maternal/fetal transfer was occurred

## References

[1] Flegel W.A., Natanson C., Klein H.G. (2014). Does prolonged storage of red blood cells cause harm?. Br. J. Haematol..

[2] Chen J.Y., Scerbo M., Kramer G. (2009). A review of blood substitutes: Examining the history, clinical trial results, and ethics of hemoglobin-based oxygen carriers. Clinics (Sao Paulo).

[3] Jahr J.S., Akha A.S., Holtby R.J. (2012). Crosslinked, polymerized, and peg-conjugated hemoglobin-based oxygen carriers: Clinical safety and efficacy of recent and current products. Curr. Drug Discov. Technol..

[4] Jahr J.S., Moallempour M., Lim J.C. (2008). Hboc-201, hemoglobin glutamer-250 (bovine), hemopure (biopure corporation). Expert Opin. Biol. Ther..

[5] Savitsky J.P., Doczi J., Black J., Arnold J.D. (1978). A clinical safety trial of stroma-free hemoglobin. Clin. Pharmacol. Ther..

[6] Buehler P.W., D’Agnillo F., Schaer D.J. (2010). Hemoglobin-based oxygen carriers: From mechanisms of toxicity and clearance to rational drug design. Trends Mol. Med..

[7] Bunn H.F., Esham W.T., Bull R.W. (1969). The renal handling of hemoglobin. I. Glomerular filtration. J. Exp. Med..

[8] Kristiansen M., Graversen J.H., Jacobsen C., Sonne O., Hoffman H.J., Law S.K., Moestrup S.K. (2001). Identification of the haemoglobin scavenger receptor. Nature.

[9] Looker D., Abbott-Brown D., Cozart P., Durfee S., Hoffman S., Mathews A.J., Miller-Roehrich J., Shoemaker S., Trimble S., Fermi G. (1992). A human recombinant haemoglobin designed for use as a blood substitute. Nature.

[10] Viele M.K., Weiskopf R.B., Fisher D. (1997). Recombinant human hemoglobin does not affect renal function in humans: Analysis of safety and pharmacokinetics. Anesthesiology.

[11] Hayes J.K., Stanley T.H., Lind G.H., East K., Smith B., Kessler K. (2001). A double-blind study to evaluate the safety of recombinant human hemoglobin in surgical patients during general anesthesia. J. Cardiothorac. Vasc. Anesth..

[12] Hermann J., Corso C., Messmer K.F. (2007). Resuscitation with recombinant hemoglobin rHB2.0 in a rodent model of hemorrhagic shock. Anesthesiology.

[13] Raat N.J., Liu J.F., Doyle M.P., Burhop K.E., Klein J., Ince C. (2005). Effects of recombinant-hemoglobin solutions rHB2.0 and rHB1.1 on blood pressure, intestinal blood flow, and gut oxygenation in a rat model of hemorrhagic shock. J. Lab. Clin. Med..

[14] Marquardt D.A., Doyle M.P., Davidson J.S., Epp J.K., Aitken J.F., Lemon D.D., Anthony-Cahill S.J. (2012). Monodisperse 130 KDa and 260 KDa recombinant human hemoglobin polymers as scaffolds for protein engineering of hemoglobin-based oxygen carriers. J. Funct. Biomater..

[15] Freytag J.W., Caspari R.F., Gorczynski R.J. (1998). Chapter 5—Recent progress in the development of recombinant human hemoglobin (rHB1.1) as an oxygen therapeuti. Blood Substitutes, Present and Future Perspectives.

[16] Kim H.W., Greenburg A.G. (2004). Artificial oxygen carriers as red blood cell substitutes: A selected review and current status. Artif. Organs.

[17] Keipert P.E., Gomez C.L., Gonzales A., Macdonald V.W., Winslow R.M. (1992). The role of the kidneys in the excretion of chemically modified hemoglobins. Biomater. Artif. Cells Immobil. Biotechnol..

[18] Benesch R., Triner L., Benesch R.E., Kwong S., Verosky M. (1984). Enhanced oxygen unloading by an interdimerically crosslinked hemoglobin in an isolated perfused rabbit heart. Proc. Natl. Acad. Sci. USA.

[19] Van der Plas J., Bleeker W.K., de Vries-van Rossen A., van Hamersveld A., Rigter G., Loos J.A., Bakker J.C. (1985). Oxygen affinity of hemoglobin solutions modified by coupling with NFPLP and the effects on tissue oxygenation in the isolated perfused rat liver. Adv. Exp. Med. Biol..

[20] Van der Plas J., de Vries-van Rossen A., Bleeker W.K., Bakker J.C. (1986). Effect of coupling of 2-nor-2-formylpyridoxal 5′-phosphate to stroma-free hemoglobin on oxygen affinity and tissue oxygenation. Studies in the isolated perfused rat liver under conditions of normoxia and stagnant hypoxia. J. Lab. Clin. Med..

[21] Bleeker W.K., van der Plas J., Agterberg J., Rigter G., Bakker J.C. (1986). Prolonged vascular retention of a hemoglobin solution modified by cross-linking with 2-nor-2-formylpyridoxal 5′-phosphate. J. Lab. Clin. Med..

[22] Keipert P.E., Verosky M., Triner L. (1989). Plasma retention and metabolic fate of hemoglobin modified with an interdimeric covalent cross link. ASAIO Trans..

[23] Bleeker W.K., van der Plas J., Feitsma R.I., Agterberg J., Rigter G., de Vries-van Rossen A., Pauwels E.K., Bakker J.C. (1989). In vivo distribution and elimination of hemoglobin modified by intramolecular cross-linking with 2-nor-2-formylpyridoxal 5′-phosphate. J. Lab. Clin. Med..

[24] Keipert P.E., Gonzales A., Gomez C.L., MacDonald V.W., Hess J.R., Winslow R.M. (1993). Acute changes in systemic blood pressure and urine output of conscious rats following exchange transfusion with diaspirin-crosslinked hemoglobin solution. Transfusion (Paris).

[25] Keipert P.E., Gomez C.L., Gonzales A., MacDonald V.W., Hess J.R., Winslow R.M. (1994). Diaspirin cross-linked hemoglobin: Tissue distribution and long-term excretion after exchange transfusion. J. Lab. Clin. Med..

[26] D’Almeida M.S., Sibbald W.J., White M., Chin-Yee I.H. (1998). Influence of sepsis on the plasma elimination pharmacokinetics of diaspirin crosslinked hemoglobin in rats. Artif. Cells Blood Substit. Immobil. Biotechnol..

[27] Palaparthy R., Kastrissios H., Gulati A. (2001). Pharmacokinetics of diaspirin cross-linked haemoglobin in a rat model of hepatic cirrhosis. J. Pharm. Pharmacol..

[28] Przybelski R.J., Daily E.K., Kisicki J.C., Mattia-Goldberg C., Bounds M.J., Colburn W.A. (1996). Phase I study of the safety and pharmacologic effects of diaspirin cross-linked hemoglobin solution. Crit. Care Med..

[29] Swan S.K., Halstenson C.E., Collins A.J., Colburn W.A., Blue J., Przybelski R.J. (1995). Pharmacologic profile of diaspirin cross-linked hemoglobin in hemodialysis patients. Am. J. Kidney Dis..

[30] Lamy M.L., Daily E.K., Brichant J.F., Larbuisson R.P., Demeyere R.H., Vandermeersch E.A., Lehot J.J., Parsloe M.R., Berridge J.C., Sinclair C.J. (2000). Randomized trial of diaspirin cross-linked hemoglobin solution as an alternative to blood transfusion after cardiac surgery. Anesthesiology.

[31] O’Hara J.F., Colburn W.A., Tetzlaff J.E., Novick A.C., Angermeier K.W., Schubert A. (2001). Hemoglobin and methemoglobin concentrations after large-dose infusions of diaspirin cross-linked hemoglobin. Anesth. Analg..

[32] Rentko V.T., Pearce L.B., Moon-Massat P.F., Gawryl M.S. (2006). Hemopure^®^ (HBOC-201, hemoglobin glutamer-250 (bovine)). Preclinical studies. Blood Substitutes.

[33] Arnaud F., Hammett M., Asher L., Philbin N., Rice J., Dong F., Pearce B., Flournoy W.S., Nicholson C., McCarron R. (2005). Effects of bovine polymerized hemoglobin on coagulation in controlled hemorrhagic shock in swine. Shock.

[34] Pearce L.B., Gawryl M.S., Rentko V.T., Moon-Massat P.F., Rausch C.W. (2006). Hboc-201 (hemoglobin glutamer-250 (bovine), hemopure^®^). Clinical studies. Blood substitutes.

[35] Hughes G.S., Antal E.J., Locker P.K., Francom S.F., Adams W.J., Jacobs E.E. (1996). Physiology and pharmacokinetics of a novel hemoglobin-based oxygen carrier in humans. Crit. Care Med..

[36] Hughes G.S., Yancey E.P., Albrecht R., Locker P.K., Francom S.F., Orringer E.P., Antal E.J., Jacobs E.E. (1995). Hemoglobin-based oxygen carrier preserves submaximal exercise capacity in humans. Clin. Pharmacol. Ther..

[37] Hughes G.S., Francome S.F., Antal E.J., Adams W.J., Locker P.K., Yancey E.P., Jacobs E.E. (1995). Hematologic effects of a novel hemoglobin-based oxygen carrier in normal male and female subjects. J. Lab. Clin. Med..

[38] Standl T., Burmeister M.A., Horn E.P., Wilhelm S., Knoefel W.T., Schulte am Esch J. (1998). Bovine haemoglobin-based oxygen carrier for patients undergoing haemodilution before liver resection. Br. J. Anaesth..

[39] Lok C. (2001). Blood product from cattle wins approval for use in humans. Nature.

[40] Mer M., Hodgson E., Wallis L., Jacobson B., Levien L., Snyman J., Sussman M.J., James M., van Gelder A., Allgaier R. (2016). Hemoglobin glutamer-250 (bovine) in south africa: Consensus usage guidelines from clinician experts who have treated patients. Transfusion (Paris).

[41] Scatena R., Giardina B. (2001). O-raffinose-polymerised haemoglobin. A biochemical and pharmacological profile of an oxygen carrier. Expert Opin. Biol. Ther..

[42] Hsia J.C., Song D.L., Er S.S., Wong L.T., Keipert P.E., Gomez C.L., Gonzales A., Macdonald V.W., Hess J.R., Winslow R.M. (1992). Pharmacokinetic studies in the rat on a O-raffinose polymerized human hemoglobin. Biomater. Artif. Cells Immobil. Biotechnol..

[43] Song S.E., David W., Lawrence T.W. (1996). Pharmacokinetics of 3h-hemolinktm in rats. Artif. Cells Blood Substit. Biotechnol..

[44] Wicks D., Wong L.T., Sandhu R., Stewart R.K., Biro G.P. (2003). The intravascular persistence and methemoglobin formation of hemolink (hemoglobin raffimer) in dogs. Artif. Cells Blood Substit. Immobil. Biotechnol..

[45] Carmichael F.J., Ali A.C., Campbell J.A., Langlois S.F., Biro G.P., Willan A.R., Pierce C.H., Greenburg A.G. (2000). A phase I study of oxidized raffinose cross-linked human hemoglobin. Crit. Care Med..

[46] Cheng D.C., Mazer C.D., Martineau R., Ralph-Edwards A., Karski J., Robblee J., Finegan B., Hall R.I., Latimer R., Vuylsteke A. (2004). A phase ii dose-response study of hemoglobin raffimer (hemolink) in elective coronary artery bypass surgery. J. Thorac. Cardiovasc. Surg..

[47] Greenburg A.G., Kim H.W. (2004). Use of an oxygen therapeutic as an adjunct to intraoperative autologous donation to reduce transfusion requirements in patients undergoing coronary artery bypass graft surgery. J. Am. Coll. Surg..

[48] Hill S.E., Gottschalk L.I., Grichnik K. (2002). Safety and preliminary efficacy of hemoglobin raffimer for patients undergoing coronary artery bypass surgery. J. Cardiothorac. Vasc. Anesth..

[49] Gould S.A., Moore E.E., Hoyt D.B., Ness P.M., Norris E.J., Carson J.L., Hides G.A., Freeman I.H., DeWoskin R., Moss G.S. (2002). The life-sustaining capacity of human polymerized hemoglobin when red cells might be unavailable. J. Am. Coll. Surg..

[50] Profile A.R.D. (2003). Human haemoglobin-northfield. Biodrugs.

[51] Sehgal L.R., Gould S.A., Rosen A.L., Sehgal H.L., Moss G.S. (1984). Polymerized pyridoxylated hemoglobin: A red cell substitute with normal oxygen capacity. Surgery.

[52] Gould S.A., Moore E.E., Hoyt D.B., Burch J.M., Haenel J.B., Garcia J., DeWoskin R., Moss G.S. (1998). The first randomized trial of human polymerized hemoglobin as a blood substitute in acute trauma and emergent surgery. J. Am. Coll. Surg..

[53] Vandegriff K.D., Winslow R.M. (2009). Hemospan: Design principles for a new class of oxygen therapeutic. Artif. Organs.

[54] Vandegriff K.D., Malavalli A., Wooldridge J., Lohman J., Winslow R.M. (2003). Mp4, a new nonvasoactive peg-hb conjugate. Transfusion (Paris).

[55] Young M.A., Malavalli A., Winslow N., Vandegriff K.D., Winslow R.M. (2007). Toxicity and hemodynamic effects after single dose administration of malpeg-hemoglobin (mp4) in rhesus monkeys. Transl. Res..

[56] Bjorkholm M., Fagrell B., Przybelski R., Winslow N., Young M., Winslow R.M. (2005). A phase I single blind clinical trial of a new oxygen transport agent (mp4), human hemoglobin modified with maleimide-activated polyethylene glycol. Haematologica.

[57] Olofsson C., Nygards E.B., Ponzer S., Fagrell B., Przybelski R., Keipert P.E., Winslow N., Winslow R.M. (2008). A randomized, single-blind, increasing dose safety trial of an oxygen-carrying plasma expander (hemospan) administered to orthopaedic surgery patients with spinal anaesthesia. Transfus. Med..

[58] Olofsson C., Ahl T., Johansson T., Larsson S., Nellgard P., Ponzer S., Fagrell B., Przybelski R., Keipert P., Winslow N. (2006). A multicenter clinical study of the safety and activity of maleimide-polyethylene glycol-modified hemoglobin (hemospan) in patients undergoing major orthopedic surgery. Anesthesiology.

[59] Conover C.D., Malatesta P., Lejeune L., Chang C.L., Shorr R.G. (1996). The effects of hemodilution with polyethylene glycol bovine hemoglobin (PEG-Hb) in a conscious porcine model. J. Investig. Med..

[60] Song D., Olano M., Wilson D.F., Pastuszko A., Tammela O., Nho K., Shorr R.G. (1995). Comparison of the efficacy of blood and polyethylene glycol-hemoglobin in recovery of newborn piglets from hemorrhagic hypotension: Effect on blood pressure, cortical oxygen, and extracellular dopamine in the brain. Transfusion (Paris).

[61] Conover C.D., Linberg R., Gilbert C.W., Shum K.L., Shorr R.G. (1997). Effect of polyethylene glycol conjugated bovine hemoglobin in both top-load and exchange transfusion rat models. Artif. Organs.

[62] Conover C.D., Linberg R., Shum K.L., Shorr R.G. (1999). The ability of polyethylene glycol conjugated bovine hemoglobin (PEG-Hb) to adequately deliver oxygen in both exchange transfusion and top-loaded rat models. Artif. Cells Blood Substit. Immobil. Biotechnol..

[63] Conover C.D., Gilbert C.W., Shum K.L., Shorr R.G. (1997). The impact of polyethylene glycol conjugation on bovine hemoglobin’s circulatory half-life and renal effects in a rabbit top-loaded transfusion model. Artif. Organs.

[64] Conover C.D., Lejeune L., Shum K., Gilbert C., Shorr R.G. (1997). Physiological effect of polyethylene glycol conjugation on stroma-free bovine hemoglobin in the conscious dog after partial exchange transfusion. Artif. Organs.

[65] Nho K., Glower D., Bredehoeft S., Shankar H., Shorr R., Abuchowski A. (1992). Peg-bovine hemoglobin: Safety in a canine dehydrated hypovolemic-hemorrhagic shock model. Biomater. Artif. Cells Immobil. Biotechnol..

[66] Ji H.J., Chai H.Y., Nahm S.S., Lee J., Bae G.W., Nho K., Kim Y.B., Kang J.K. (2007). Neuroprotective effects of the novel polyethylene glycol-hemoglobin conjugate SB1 on experimental cerebral thromboembolism in rats. Eur. J. Pharmacol..

[67] Lee J., Lee J., Yoon S., Nho K. (2006). Pharmacokinetics of 125I-radiolabelled PEG-hemoglobin SB1. Artif. Cells Blood Substit. Immobil. Biotechnol..

[68] Kwon O.S., Chung U.T., Chung Y.B. (2004). Pharmacokinetics of PEG-hemoglobin SB1, a hemoglobin-based oxygen carrier, after its intravenous administration in beagle dogs. Arch. Pharm. Res..

[69] Natanson C., Kern S.J., Lurie P., Banks S.M., Wolfe S.M. (2008). Cell-free hemoglobin-based blood substitutes and risk of myocardial infarction and death: A meta-analysis. JAMA.

[70] Njoku M., St Peter D., Mackenzie C.F. (2015). Haemoglobin-based oxygen carriers: Indications and future applications. Br. J. Hosp. Med. (Lond.).

[71] Razynska A., Bucci E. (1998). Chapter 21—Zero-link polymerization: A new class of polymeric hemoglobins. Blood Substitutes, Present and Future Perspectives.

[72] Harrington J.P., Wollocko H., Kim H.W., Greenburg A.G. (2013). Zero-link hemoglobin (oxyvita^®^): Impact of molecular design characteristics on pre-clinical studies. Hemoglobin-Based Oxygen Carriers as Red Cell Substitutes and Oxygen Therapeutics.

[73] Harrington J.P., Wollocko H. (2011). Molecular design properties of oxyvita hemoglobin, a new generation therapeutic oxygen carrier: A review. J. Funct. Biomater..

[74] Wollocko H., Anvery S., Wollocko J., Harrington J.M., Harrington J.P. (2016). Zero-link polymerized hemoglobin (OxyVita®Hb) stabilizes the heme environment: Potential for lowering vascular oxidative stress. Artif. Cells Nanomed. Biotechnol..

[75] Harrington J.P., Wollocko H. (2010). Pre-clinical studies using oxyvita hemoglobin, a zero-linked polymeric hemoglobin: A review. J. Artif. Organs.

[76] Harrington J.P., Wollocko J., Kostecki E., Wollocko H. (2011). Physicochemical characteristics of oxyvita hemoglobin, a zero-linked polymer: Liquid and powder preparations. Artif. Cells Blood Substit. Immobil. Biotechnol..

[77] Matheson B., Kwansa H.E., Bucci E., Rebel A., Koehler R.C. (2002). Vascular response to infusions of a nonextravasating hemoglobin polymer. J. Appl. Physiol..

[78] Abuchowski A. (2016). Pegylated bovine carboxyhemoglobin (sanguinate): Results of clinical safety testing and use in patients. Adv. Exp. Med. Biol..

[79] Ananthakrishnan R., Li Q., O’Shea K.M., Quadri N., Wang L., Abuchowski A., Schmidt A.M., Ramasamy R. (2013). Carbon monoxide form of pegylated hemoglobin protects myocardium against ischemia/reperfusion injury in diabetic and normal mice. Artif. Cells Nanomed. Biotechnol..

[80] Mullah S.H., Abutarboush R., Moon-Massat P.F., Saha B.K., Haque A., Walker P.B., Auker C.R., Arnaud F.G., McCarron R.M., Scultetus A.H. (2016). Sanguinate’s effect on pial arterioles in healthy rats and cerebral oxygen tension after controlled cortical impact. Microvasc. Res..

[81] Zhang J., Cao S., Kwansa H., Crafa D., Kibler K.K., Koehler R.C. (2012). Transfusion of hemoglobin-based oxygen carriers in the carboxy state is beneficial during transient focal cerebral ischemia. J. Appl. Physiol..

[82] Misra H., Lickliter J., Kazo F., Abuchowski A. (2014). Pegylated carboxyhemoglobin bovine (sanguinate): Results of a phase i clinical trial. Artif. Organs.

[83] Misra H., Bainbridge J., Berryman J., Abuchowski A., Galvez K.M., Uribe L.F., Hernandez A.L., Sosa N.R. (2017). A phase Ib open label, randomized, safety study of SANGUINATE™ in patients with sickle cell anemia. Rev. Bras. Hematol. Hemoter..

[84] Schallner N., Pandit R., LeBlanc R., Thomas A.J., Ogilvy C.S., Zuckerbraun B.S., Gallo D., Otterbein L.E., Hanafy K.A. (2015). Microglia regulate blood clearance in subarachnoid hemorrhage by heme oxygenase-1. J. Clin. Investig..

[85] Vandegriff K.D., Young M.A., Lohman J., Bellelli A., Samaja M., Malavalli A., Winslow R.M. (2008). Co-mp4, a polyethylene glycol-conjugated haemoglobin derivative and carbon monoxide carrier that reduces myocardial infarct size in rats. Br. J. Pharmacol..

[86] Belcher J.D., Young M., Chen C., Nguyen J., Burhop K., Tran P., Vercellotti G.M. (2013). MP4CO, a pegylated hemoglobin saturated with carbon monoxide, is a modulator of HO-1, inflammation, and vaso-occlusion in transgenic sickle mice. Blood.

[87] Keipert P.E. (2016). Clinical evaluation of MP4CO: A phase 1b escalating-dose, safety and tolerability study in stable adult patients with sickle cell disease. Adv. Exp. Med. Biol..

[88] Tomita D., Kimura T., Hosaka H., Daijima Y., Haruki R., Ludwig K., Bottcher C., Komatsu T. (2013). Covalent core-shell architecture of hemoglobin and human serum albumin as an artificial O_2_ carrier. Biomacromolecules.

[89] Haruki R., Kimura T., Iwasaki H., Yamada K., Kamiyama I., Kohno M., Taguchi K., Nagao S., Maruyama T., Otagiri M. (2015). Safety evaluation of hemoglobin-albumin cluster "HemoAct" as a red blood cell substitute. Sci. Rep..

[90] Yamada K., Yokomaku K., Haruki R., Taguchi K., Nagao S., Maruyama T., Otagiri M., Komatsu T. (2016). Influence of molecular structure on O_2_-binding properties and blood circulation of hemoglobinalbumin clusters. PLoS ONE.

[91] Komatsu T., Oguro Y., Nakagawa A., Tsuchida E. (2005). Albumin clusters: Structurally defined protein tetramer and oxygen carrier including thirty-two iron(II) porphyrins. Biomacromolecules.

[92] Hosaka H., Haruki R., Yamada K., Bottcher C., Komatsu T. (2014). Hemoglobin-albumin cluster incorporating a pt nanoparticle: Artificial O_2_ carrier with antioxidant activities. PLoS ONE.

[93] Adamantos S., Boag A., Hughes D. (2005). Clinical use of a haemoglobin-based oxygen-carrying solution in dogs and cats. In Pract..

[94] Yamada K., Yokomaku K., Kureishi M., Akiyama M., Kihira K., Komatsu T. (2016). Artificial blood for dogs. Sci. Rep..

[95] Sakai H., Sou K., Horinouchi H., Kobayashi K., Tsuchida E. (2009). Review of hemoglobin-vesicles as artificial oxygen carriers. Artif. Organs.

[96] Sakai H., Sou K., Horinouchi H., Kobayashi K., Tsuchida E. (2008). Haemoglobin-vesicles as artificial oxygen carriers: Present situation and future visions. J. Intern. Med..

[97] Kaneda S., Ishizuka T., Goto H., Kimura T., Inaba K., Kasukawa H. (2009). Liposome-encapsulated hemoglobin, TRM-645: Current status of the development and important issues for clinical application. Artif. Organs.

[98] Yoshioka H. (1991). Surface modification of haemoglobin-containing liposomes with polyethylene glycol prevents liposome aggregation in blood plasma. Biomaterials.

[99] Goto H., Ogata Y., Suzuki K., Kamitani T. (1995). Large scale production of neo red cell. Aritif. Blood.

[100] Usuba A., Osuka F., Kimura T., Sato R., Ogata Y., Gotoh H., Kimura T., Fukui H. (1998). Effect of liposome-encapsulated hemoglobin, neo red cells, on hemorrhagic shock. Surg. Today.

[101] Kiwada H., Matsuo H., Harashima H. (1998). Identification of proteins mediating clearance of liposomes using a liver perfusion system. Adv. Drug Deliv. Rev..

[102] Taguchi K., Urata Y., Anraku M., Maruyama T., Watanabe H., Sakai H., Horinouchi H., Kobayashi K., Tsuchida E., Kai T. (2009). Pharmacokinetic study of enclosed hemoglobin and outer lipid component after the administration of hemoglobin vesicles as an artificial oxygen carrier. Drug Metab. Dispos..

[103] Sou K., Klipper R., Goins B., Tsuchida E., Phillips W.T. (2005). Circulation kinetics and organ distribution of HB-vesicles developed as a red blood cell substitute. J. Pharmacol. Exp. Ther..

[104] Taguchi K., Watanabe H., Sakai H., Horinouchi H., Kobayashi K., Maruyama T., Otagiri M. (2012). A fourteen-day observation and pharmacokinetic evaluation after a massive intravenous infusion of hemoglobin-vesicles (artificial oxygen carriers) in cynomolgus monkeys. J. Drug Metab. Toxicol..

[105] Sakai H., Masada Y., Horinouchi H., Yamamoto M., Ikeda E., Takeoka S., Kobayashi K., Tsuchida E. (2004). Hemoglobin-vesicles suspended in recombinant human serum albumin for resuscitation from hemorrhagic shock in anesthetized rats. Crit. Care Med..

[106] Yamamoto M., Horinouchi H., Kobayashi K., Seishi Y., Sato N., Itoh M., Sakai H. (2012). Fluid resuscitation of hemorrhagic shock with hemoglobin vesicles in beagle dogs: Pilot study. Artif. Cells Blood Substit. Immobil. Biotechnol..

[107] Seishi Y., Horinouchi H., Sakai H., Kobayashi K. (2012). Effect of the cellular-type artificial oxygen carrier hemoglobin vesicle as a resuscitative fluid for prehospital treatment: Experiments in a rat uncontrolled hemorrhagic shock model. Shock.

[108] Tokuno M., Taguchi K., Yamasaki K., Sakai H., Otagiri M. (2016). Long-term stored hemoglobin-vesicles, a cellular type of hemoglobin-based oxygen carrier, has resuscitative effects comparable to that for fresh red blood cells in a rat model with massive hemorrhage without post-transfusion lung injury. PLoS ONE.

[109] Taguchi K., Maruyama T., Iwao Y., Sakai H., Kobayashi K., Horinouchi H., Tsuchida E., Kai T., Otagiri M. (2009). Pharmacokinetics of single and repeated injection of hemoglobin-vesicles in hemorrhagic shock rat model. J. Control. Release.

[110] Taguchi K., Maruyama T., Otagiri M. (2011). Pharmacokinetic properties of hemoglobin vesicles as a substitute for red blood cells. Drug Metab. Rev..

[111] Taguchi K., Miyasato M., Watanabe H., Sakai H., Tsuchida E., Horinouchi H., Kobayashi K., Maruyama T., Otagiri M. (2011). Alteration in the pharmacokinetics of hemoglobin-vesicles in a rat model of chronic liver cirrhosis is associated with kupffer cell phagocyte activity. J. Pharm. Sci..

[112] Kaga M., Li H., Ohta H., Taguchi K., Ogaki S., Izumi H., Inagaki M., Tsuchiya S., Okamura K., Otagiri M. (2012). Liposome-encapsulated hemoglobin (hemoglobin-vesicle) is not transferred from mother to fetus at the late stage of pregnancy in the rat model. Life Sci..

[113] Taguchi K., Nagao S., Yamasaki K., Sakai H., Seo H., Maruyama T., Otagiri M. (2015). Biological responsiveness and metabolic performance of liposome-encapsulated hemoglobin (hemoglobin-vesicles) in apolipoprotein e-deficient mice after massive intravenous injection. Biol. Pharm. Bull..

[114] Takaori M. (2005). Interpretation of a guidance for oxygen carrier products and their manufacturing: Proposed by the society of blood substitutes japan. Artif. Blood.

[115] Li H., Ohta H., Tahara Y., Nakamura S., Taguchi K., Nakagawa M., Oishi Y., Goto Y., Wada K., Kaga M. (2015). Artificial oxygen carriers rescue placental hypoxia and improve fetal development in the rat pre-eclampsia model. Sci. Rep..

[116] Nagao S., Taguchi K., Miyazaki Y., Wakayama T., Chuang V.T., Yamasaki K., Watanabe H., Sakai H., Otagiri M., Maruyama T. (2016). Evaluation of a new type of nano-sized carbon monoxide donor on treating mice with experimentally induced colitis. J. Control. Release.

[117] Nagao S., Taguchi K., Sakai H., Yamasaki K., Watanabe H., Otagiri M., Maruyama T. (2016). Carbon monoxide-bound hemoglobin vesicles ameliorate multiorgan injuries induced by severe acute pancreatitis in mice by their anti-inflammatory and antioxidant properties. Int. J. Nanomed..

[118] Nagao S., Taguchi K., Sakai H., Tanaka R., Horinouchi H., Watanabe H., Kobayashi K., Otagiri M., Maruyama T. (2014). Carbon monoxide-bound hemoglobin-vesicles for the treatment of bleomycin-induced pulmonary fibrosis. Biomaterials.

[119] Araki J., Sakai H., Takeuchi D., Kagaya Y., Tashiro K., Naito M., Mihara M., Narushima M., Iida T., Koshima I. (2015). Normothermic preservation of the rat hind limb with artificial oxygen-carrying hemoglobin vesicles. Transplantation.

[120] Taguchi K., Miyasato M., Ujihira H., Watanabe H., Kadowaki D., Sakai H., Tsuchida E., Horinouchi H., Kobayashi K., Maruyama T. (2010). Hepatically-metabolized and -excreted artificial oxygen carrier, hemoglobin vesicles, can be safely used under conditions of hepatic impairment. Toxicol. Appl. Pharmacol..

[121] Estep T.N. (2015). Pharmacokinetics and mechanisms of plasma removal of hemoglobin-based oxygen carriers. Artif. Cells Nanomed. Biotechnol..

